# Impact of Farm Management Practices on *Salmonella* Occurrence at the Farm Level—A Blend of Traditional Methods and Artificial Intelligence

**DOI:** 10.3390/foods15040676

**Published:** 2026-02-12

**Authors:** Diana Marcu, Igori Balta, Michael Harvey, David McCleery, Adela Marcu, Gratiela Gradisteanu-Pircalabioru, Todd Callaway, Tiberiu Iancu, Ioan Pet, Florica Morariu, Ana-Maria Imbrea, Gabi Dumitrescu, Liliana Petculescu Ciochina, Lavinia Stef, Nicolae Corcionivoschi

**Affiliations:** 1 Faculty of Bioengineering of Animal Resources, University of Life Sciences King Mihai I from Timisoara, 300645 Timisoara, Romania; diana.marcu@usvt.ro (D.M.); balta.igori@usvt.ro (I.B.); david.mccleery@afbini.gov.uk (D.M.); adelamarcu@usvt.ro (A.M.); ioanpet@usvt.ro (I.P.); floricamorariu@usvt.ro (F.M.); anamaria.imbrea@usvt.ro (A.-M.I.); gabidumitrescu@usvt.ro (G.D.); lilianapetculescuciochina@usvt.ro (L.P.C.); laviniastef@usvt.ro (L.S.); 2 Bacteriology Branch, Veterinary Sciences Division, Agri-Food and Biosciences Institute, Belfast BT4 3SD, Northern Ireland, UK; michael.harvey@afbini.gov.uk; 3 Faculty of Biology, University of Bucharest, 050097 Bucharest, Romania; gratiela.gradisteanu@icub.unibuc.ro; 4 Department of Animal and Dairy Science, University of Georgia, Athens, GA 30602, USA; todd.callaway@uga.edu; 5 Faculty of Management and Rural Development, University of Life Sciences King Mihai I from Timisoara, 300645 Timisoara, Romania; tiberiuiancu@usvt.ro; 6 Academy of Romanian Scientists, 050044 Bucharest, Romania

**Keywords:** *Salmonella*, farm management, artificial intelligence (AI), poultry, pigs, dairy

## Abstract

**Background**: *Salmonella enterica* remains a leading cause of foodborne illness worldwide despite decades of advances in surveillance and control. Traditional interventions have targeted specific points in the food chain, yet recurrent outbreaks show that *Salmonella* exploits system-wide gaps and inconsistencies. **Methods**: This review synthesises recent evidence from epidemiology, experimental microbiology, and regulatory practice to evaluate how management decisions, from farm through processing, influence *Salmonella* risk in livestock-derived foods. **Results**: Poultry, pig, and cattle farms employ targeted measures, including rodent control, litter management, batch rearing, and secure feed storage, to reduce contamination. The greatest reductions in *Salmonella* prevalence occur when these measures are embedded in coherent farm-to-fork programmes. Future gains are likely to come less from novel interventions and more from rigorous implementation, integration, and the validation of existing tools, supported by high-resolution surveillance (including whole-genome sequencing) and prevention-focused management systems. Artificial intelligence can enhance control through real-time surveillance, predictive risk modelling, and targeted interventions informed by diverse farm data. **Conclusions**: Sustained progress in *Salmonella* control will depend on rigorously applying existing interventions, supported by high-resolution surveillance and prevention-focused management. Carefully governed AI can enhance real-time monitoring and risk prediction, but its value hinges on addressing data, cost, and regulatory challenges.

## 1. Introduction

Non-typhoidal *Salmonella enterica* (NTS) is a leading foodborne pathogen worldwide, causing acute gastroenteritis and, in vulnerable hosts, invasive disease. Recent global burden estimates suggest that NTS results in approximately 90–100 million episodes of enterocolitis each year and around 50,000–160,000 deaths, depending on the modelling framework used. Some analyses combining diarrhoeal and invasive disease suggest up to ~290,000 deaths annually [1,2]. In the European Union (EU) alone, over 91,000 human salmonellosis cases are reported each year, with an estimated economic cost of up to €3 billion. In 2018, *Salmonella* accounted for more than half of all foodborne outbreak-related illnesses [3,4]. Across the EU, source-attribution models indicate that eggs and laying hens account for approximately 40–45% of human salmonellosis cases, pigs for around 30%, and pork products about 9% of outbreaks—making pork the most significant meat source of *Salmonella* for humans [2,5]. Contamination by *Salmonella enterica* is most often linked to foods of animal origin—especially poultry, pork, and eggs—but also occurs in produce, dairy, and other products. The burden is also considerable in the United States, where *Salmonella* remains a leading cause of foodborne bacterial illness. Current estimates suggest that nontyphoidal *Salmonella* causes about 1.35 million illnesses, tens of thousands of hospitalisations, and several hundred deaths each year, with an associated economic cost of roughly US$4.1 billion annually (2018 dollars) [6]. Historically, human salmonellosis has been primarily linked to foods of animal origin—particularly poultry, eggs, pork, and beef—and to contaminated animal feed. However, outbreaks linked to fresh produce, dairy, and other commodities are increasingly being recognised [4]. The consumption of unpasteurised milk or cheese has repeatedly caused large multi-state outbreaks in North America and Europe [7].

Epidemiologically, NTS has a broad ecological niche. It comprises thousands of serovars capable of infecting a wide range of hosts, and many serovars colonise the intestinal tract of livestock and wildlife with limited or no clinical signs, enabling silent on-farm amplification and environmental shedding [1]. *Salmonella* can persist in farm environments (soil, water, litter, manure) and in food-processing facilities, where it survives on equipment surfaces and in biofilms for prolonged periods, particularly when sanitation is suboptimal [8,9]. This ecological versatility underpins its ability to contaminate a wide range of products, including fresh produce, dairy products, spices, and low-moisture foods [4]. Consequently, controlling *Salmonella* throughout the food chain is now recognised as a key One Health priority, requiring coordinated measures from primary production to processing and retail. Since the 1990s, many countries have introduced national *Salmonella* control programmes targeting primary production and slaughter/processing [1]. The adoption of Good Manufacturing Practices (GMPs) and Standard Operating Procedures (SOPs) is also fundamental in industry. These programmes combine on-farm measures (e.g., biosecurity, vaccination, feed and water hygiene, rodent control) with processing-level measures (e.g., scalding and carcass decontamination, equipment sanitation in food industries) [8]. Management practices at both the farm and processing levels are therefore crucial determinants of *Salmonella* prevalence at critical control points.

**Figure 1:**
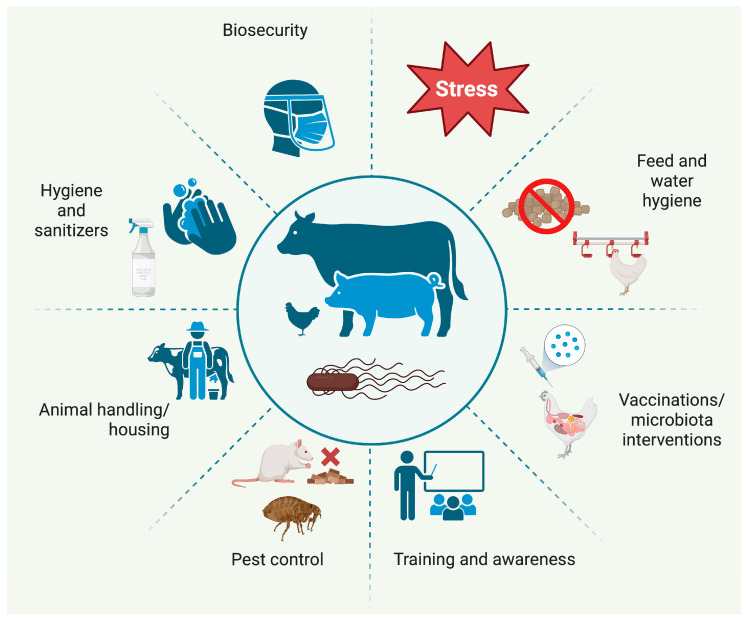
Traditional farm management practices with impact on *Salmonella* prevalence. Created with Biorender.com.

This comprehensive narrative review synthesises recent evidence from epidemiology, experimental microbiology, and food safety management to clarify how specific management practices influence *Salmonella* enterica risks along the livestock-derived food chain, with a particular focus on the European Union and the United States. Instead of merely cataloguing all available studies, we concentrated on key findings regarding the effects of on-farm interventions—such as biosecurity, vaccination, feed and manure management, and animal flow—on herd and flock prevalence (Figure 1). By integrating these aspects with AI, our objective was to identify management strategies that have effectively reduced *Salmonella* prevalence, highlight ongoing vulnerabilities that continue to cause outbreaks, and guide the design and validation of future farm-to-plant control programmes.

## 2. *Salmonella* in Farm Environments: Impact of Management Practices

Livestock production is a significant reservoir for this pathogen; poultry and pork are consistently identified as primary sources of human salmonellosis, followed by beef and eggs [1,10]. In the EU, control of *Salmonella* is a top priority due to its public health and economic impacts. Although it often causes few or no clinical signs in farm animals, *Salmonella* can colonise the gut of chickens, pigs, and cattle without causing overt clinical signs and can be shed into the farm environment [1]. If not managed at the farm level, these bacteria can contaminate animal-derived foods, as evidence shows that higher on-farm *Salmonella* prevalence is associated with greater contamination of meat carcasses at slaughter. For instance, targeted control measures in European layer hen flocks have been associated with reductions of more than 50% in human salmonellosis cases linked to eggs [11]. Effective farm-level control of *Salmonella* (pre-harvest) can substantially reduce the pathogen’s entry into the food chain. Key practices include biosecurity, hygiene and sanitation, animal handling and housing strategies, dietary and microbial interventions, and staff training.

### 2.1. Poultry Farms (Broilers and Layers)

**Figure 2:**
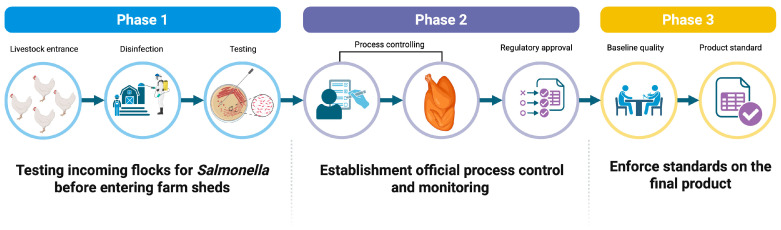
Three-phase regulatory framework to control *Salmonella* infections linked to poultry consumption. Created with Biorender.com.

Poultry, including broiler chickens, laying hens, and turkeys, is a well-recognised reservoir of *Salmonella*, and poultry products are among the most frequently implicated vehicles in foodborne outbreaks. Over the past two decades, the EU has implemented stringent control programmes for *Salmonella* in poultry, contributing to significant reductions in flock infection rates and human salmonellosis, particularly egg-associated *S. enteritidis* [11]. Achieving and maintaining these gains depends critically on meticulous on-farm management. Below, we summarise key management factors that influence *Salmonella* prevalence in poultry flocks (Figure 2).

#### 2.1.1. Biosecurity Measures

**Figure 3:**
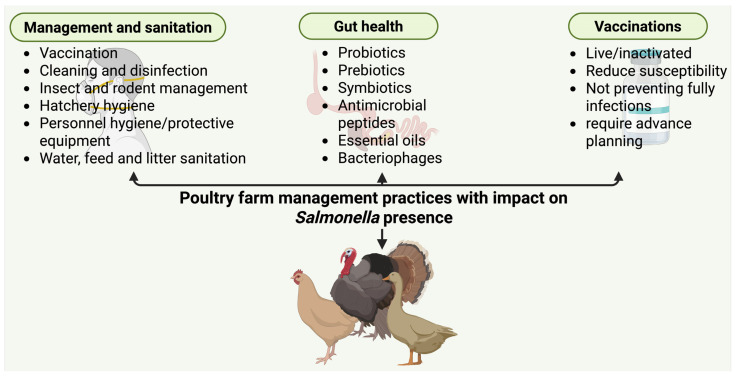
Management practices in poultry farms designed to control *Salmonella* infections. Created with Biorender.com.

Robust biosecurity is one of the most cost-effective strategies to prevent the introduction and spread of *Salmonella* on poultry farms [8]. Core biosecurity principles include restricting access to farms (limiting visitors and requiring protective clothing and boot changes), establishing physical barriers (perimeter fencing and controlled entry points), using disinfectant footbaths at house entrances, and strictly separating different poultry houses or age groups [8,12]. The use of dedicated clothing, footwear, and equipment, as well as clearly defined movement protocols for staff and visitors, plays a critical role in preventing the introduction of *Salmonella* via fomites. Vector control is vital for biosecurity. Rodents, wild birds, flies, and darkling beetles are well-known carriers of *Salmonella* into and around poultry houses. Mice and rats can shed *Salmonella* in their droppings, contaminating feed, litter, and structures; wild birds can introduce environmental strains onto free-range areas or into ventilation inlets; and beetles and flies often transfer bacteria between birds and buildings [8,12]. Several studies show that poultry farms with inadequate rodent control have significantly higher odds of *Salmonella* contamination, and rodents on *Salmonella*-positive farms often test positive for the same serovars as the flock [12,13,14]. Therefore, implementing effective pest management, including sealing and elevating feed bins, systematically baiting and trapping rodents, restricting wild bird access to houses, and controlling insects, is essential. The most common management tools used to reduce salmonellosis incidence on poultry farms are shown in Figure 3.

The detection of *Salmonella* in a farm setting triggers various management actions aimed at preventing infection in subsequent flocks. Rigorous cleaning and disinfection (C&D) between flocks, along with a strict all-in/all-out policy, are recommended to break infection cycles. In practice, C&D must be thorough: all organic material (manure, dust, feathers) should be removed before detergents and disinfectants are applied; residual organic matter markedly reduces the efficacy of disinfectants [10]. High-pressure washing is generally discouraged in poultry houses as it can aerosolise *Salmonella* and spread contamination to previously clean areas; instead, low-pressure foam or detergent application followed by rinsing and targeted disinfection is preferred [10]. Observational studies and risk-factor analyses show that farms with structured sanitation protocols and high composite biosecurity scores have lower *Salmonella* prevalence in flocks. In contrast, lapses such as uncontrolled personnel movement between houses, inadequate equipment cleaning, or ineffective rodent control are consistently associated with higher risk [12].

Quantitative evidence on specific measures in poultry remains limited, but meta-analyses in other species suggest the potential scale of impact. For example, a recent systematic review in pig herds found that farms implementing rodent control or feed/drinking-water acidification had substantially lower odds of *Salmonella* positivity (summary odds ratios ≈ 0.2–0.3), whereas all-in/all-out management showed a modest, non-significant protective effect and disinfection alone showed no clear association [15]. Although species and system differences mean that these estimates cannot be transferred directly to poultry, they support the principle that well-implemented biosecurity measures—especially rodent control, traffic control and acidified feed or water—can dramatically reduce *Salmonella* pressure. In poultry, equipment should not be shared between farms unless thoroughly cleaned and disinfected, and any new birds introduced (e.g., replacement pullets) should be sourced from monitored flocks and, ideally, quarantined or tested before mixing. In practice, effective biosecurity is not a one-size-fits-all checklist but a farm-specific system built around universal pillars of isolation, traffic control, and sanitation [10].

#### 2.1.2. Hygiene and Sanitation on Farms

Within a poultry house, hygiene practices aim to reduce environmental contamination and break the cycle of infection between flocks. A core practice is clean-out and disinfection between flocks (“all-in, all-out” management). Thorough cleaning of litter, heat-treated reuse, followed by the disinfectant treatment of floors, walls, and equipment, can significantly reduce residual *Salmonella* levels. New approaches are being explored to enhance farm sanitation, including alternative disinfectants such as essential-oil-based formulations, peroxyacids, and organic acids, as well as strategies targeting biofilm-associated *Salmonella*. To ensure efficacy, it is critical that all surfaces, including feeders, drinkers, and ventilation systems, are regularly cleaned and disinfected to remove organic matter and microbial contamination. Water lines and drinkers should be flushed and sanitised to prevent biofilm formation, which can harbour *Salmonella* and other pathogens. In addition, the use of sanitised footbaths at entry points and designated clean and dirty zones within poultry houses further reduces the risk of cross-contamination. Consistent adherence to these hygiene protocols, supported by regular staff training and monitoring, has been shown to lower *Salmonella* prevalence in poultry flocks and reduce the risk of contamination spreading throughout the production system [16].

Litter management is a crucial factor in on-farm *Salmonella* ecology. Fresh wood shavings have consistently been linked to a higher risk of *Salmonella* compared with reused, treated litter; studies have demonstrated that properly reused or composted broiler litter can lower *Salmonella* prevalence over successive cycles, likely due to competitive exclusion by a stable resident microbiota and the effects of heat and ammonia during reuse. Controlled reuse or heat-treatment of litter between flocks, rather than defaulting to fresh shavings, is increasingly recommended as a best practice. Maintaining dry, friable bedding, along with adequate ventilation to reduce moisture, further creates an environment less conducive to *Salmonella* survival and multiplication [8,17].

#### 2.1.3. Animal Handling and Housing

The management and housing of birds can influence the spread of *Salmonella*. Densely stocked or highly stressed flocks are more likely to shed additional *Salmonella*. Gentle handling and minimising stress, especially during catching and transport to slaughter, can decrease faecal shedding during transit. On poultry farms, all-in/all-out flock management (avoiding mixing birds of different ages and fully depopulating and cleaning between flocks) is recommended as a vital biosecurity measure. In a notable risk factor study of commercial laying hen farms in Great Britain, all-in/all-out operations were associated with a considerably lower risk of *S. enteritidis* infection (OR ≈ 0.06, 95% CI 0.01–0.24) [18,19]. Segregating flocks by age and preventing contact between chicks and older birds, which may be potential carriers, is standard practice. Some housing systems present challenges; for example, in layer hens, cage-free or barn systems expose birds to greater environmental contact (including contact with litter and faeces) than closed cages, which can increase *Salmonella* risk unless strict hygiene measures are implemented. Vaccination and competitive exclusion strategies are therefore even more crucial in non-cage systems. Cage-based systems confine birds to smaller areas, which historically facilitated faecal removal (in layer cages with manure belts) but also create high-density conditions and make cleaning the structure difficult. Risk factor studies have found that cage systems are at higher risk, although not all surveys agree, and flock size/management confound this relationship. The reasons include the accumulation of faecal dust in cage facilities and the complexity of effectively disinfecting cage racks and belt systems, which can harbour *Salmonella* between flocks. Researchers have found that *Salmonella* recovery rates from environmental samples (dust, faeces) tend to be higher in cage-based houses than in barn or free-range houses. In addition, older cage layer farms with deep pit manure storage can allow *Salmonella* to persist in situ for long durations, serving as a reservoir that can reinfect successive flocks [12].

Free-range and outdoor-access systems involve different risks. Birds with outdoor access may encounter contaminated soil, wild bird droppings, insects, and other environmental sources of *Salmonella*. Managing biosecurity in open systems is inherently more challenging; it is impossible to fully exclude wild birds or control all environmental contamination. Some European surveys have reported a higher exposure of free-range flocks to *Salmonella* in the range environment, yet paradoxically lower in-house contamination than in large cage complexes, likely due to smaller flock sizes, pasture rotation, and manure management practices. This inconsistency probably results from differences in farm management and size. Outdoor flocks are exposed to environmental factors, but they are often kept in smaller units or under practices (such as periodic pasture rotation) that may reduce *Salmonella* risk [12,20,21]. Smaller flock sizes, pasture rotation, and manure management influence *Salmonella* risk primarily through their effects on infection pressure, contact rates between animals and contaminated material, and pathogen persistence in the environment [22].

#### 2.1.4. Vaccination and Microbiota Interventions

Vaccination of poultry has become a vital part of controlling *Salmonella* in the EU and the UK, and its significance is increasing in the U.S. Several commercial vaccines, both live-attenuated and inactivated, are approved to target the most problematic serovars, especially *S. enteritidis* and *S. typhimurium*. The EU’s control programme for table eggs, for example, strongly encourages or mandates the vaccination of laying hens against *S. enteritidis* in many member states. Over the past decade, vaccination efforts have clearly reduced *Salmonella* infection rates in poultry. Although vaccination rarely leads to complete eradication, some field and experimental studies indicate that an effective vaccination programme can significantly decrease *Salmonella* prevalence within flocks, often achieving reductions of 20–60% in flock-level infection or egg contamination [23]. Large-scale epidemiological data support these findings: as previously noted, the deployment of *Salmonella* vaccination and other control measures in EU layer hens was associated with a greater than 50% reduction in human egg-borne salmonellosis [19], demonstrating the role of vaccines in interrupting the transmission chain from farm to table. Vaccines, both live attenuated and inactivated, targeting *S. enteritidis* and *S. typhimurium*, are widely used in layer hens, breeder flocks, and in some broiler programmes [8]. Vaccinated flocks exhibit significantly lower *Salmonella* colonisation and shedding, resulting in fewer contaminated eggs or broilers entering processing. In the UK, the introduction of comprehensive quality schemes in the late 1990s, combining hatchery hygiene, flock surveillance, improved biosecurity, and notably routine vaccination of layers, coincided with a marked decline in human *S. enteritidis* infections linked to eggs [24]. Vaccination is regarded as one of the most effective preventive measures, often providing a high return on investment through improved food safety [8]. Live attenuated *Salmonella* vaccines, usually administered via drinking water or spray, are widely used in broilers and layers because they induce local gut immunity and can provide broad protection, even against serovars that differ from the vaccine strain. They may also offer some competitive exclusion effect by temporarily colonising the intestine [12]. Current research is exploring next-generation *Salmonella* vaccines, including subunit vaccines (e.g., purified outer membrane proteins or flagella antigens) and innovative platforms such as nanoparticle-based oral vaccines. These aim to elicit strong immunity without live bacteria and may simplify administration (e.g., via feed or water). While mostly experimental to date, such vaccines could further enhance *Salmonella* control in the coming years. It is also worth noting that vaccination’s effectiveness can vary with serovar and field conditions—for instance, vaccines tend to be very effective against *S. enteritidis* in layers, but controlling *S. typhimurium* in broilers via vaccination has been more challenging. Nonetheless, the consensus in the recent literature is that vaccination, when properly applied, is a highly effective tool to reduce flock-level *Salmonella* burden. Vaccines do not eliminate *Salmonella* on their own, but they significantly raise the threshold for infection and reduce bacterial shedding, thereby complementing biosecurity and hygiene measures [12,25].

Alongside vaccination, poultry producers are increasingly using feed and water additives to modify gut microbiota and create conditions unfavourable to *Salmonella*. Organic acids (e.g., formic, propionic, and buffered blends) in feed or drinking water can reduce crop and caecal colonisation, with recent meta-analysis indicating substantial reductions in the odds of *Salmonella*-positive birds when these acids are applied in appropriate conditions [15]. Probiotics and competitive exclusion cultures, especially when administered to young chicks, can prevent or significantly limit early colonisation by occupying ecological niches and producing antimicrobials or acids [8]. Increasingly, a multi-hurdle approach combining vaccination, acidified feed or water, and early-life competitive exclusion is recommended as best practice. Integrative analyses suggest that such combinations achieve the greatest reductions in *Salmonella* loads compared with single interventions [26].

#### 2.1.5. Training and Awareness

Ultimately, all control measures on poultry farms rely on human behaviour. Training and awareness among farmers and farm staff are therefore essential. Workers must understand not only what biosecurity and hygiene procedures are necessary—for example, correct use of footbaths, respecting downtime between flocks, following movement protocols—but also why cutting corners compromises flock health and food safety. Unfortunately, multiple surveys in Europe, North America, and Asia show that the perceived benefits of biosecurity are often underestimated; farmers may regard specific measures as costly, inconvenient, or of limited impact, leading to incomplete implementation [11]. Emphasising the connection between on-farm practices and food safety outcomes is crucial. In Europe, official *Salmonella* control programmes have helped raise awareness by making *Salmonella* reduction an industry standard, whereas in regions lacking such programmes, education and incentives are needed to encourage farm-level vigilance [1]. Overall, well-managed poultry farms that implement strict biosecurity, in combination with vaccination, feed management, and environmental control in pre-harvest settings, and processing plant interventions (antimicrobials, sanitation, and quality control measures) can achieve very low *Salmonella* prevalence, thereby significantly reducing the pathogen load entering processing [8].

#### 2.1.6. Feed and Water Hygiene

Feed and water are major potential vehicles for *Salmonella* transmission into poultry flocks. Contaminated feed has historically been a common source of *Salmonella* introduction, leading to regulations on feed mill hygiene and monitoring programmes. *Salmonella* can enter feed through animal-derived ingredients (e.g., fish meal, meat-and-bone meal) or during feed processing and transport [12]. Modern feed production within the EU typically involves thermal treatments (pelleting, extrusion) that significantly reduce *Salmonella* contamination in the final product. Indeed, the heat pelleting of feed has proven effective in killing *Salmonella*, and EU regulations require feed mills to adopt HACCP-based controls to minimise contamination. Farms should source feed from suppliers that monitor *Salmonella* rigorously. On the farm, feed should be stored in closed silos to prevent contamination by rodents or wild birds. It is also advisable to periodically test feed for *Salmonella*, particularly if home-made or if there is a reason to suspect contamination [12].

Regarding nutrition, increasing evidence suggests that feed form and composition can influence *Salmonella* in the bird’s gut. Coarse mash or diets with coarsely ground or whole grains, along with selected functional feed additives, can promote gut conditions less favourable to *Salmonella* colonisation [27,28,29]. In broilers, such feeding strategies improve gizzard development and acidification, extend digesta retention, and modify caecal microbiota composition. These indirect effects are considered more significant than any direct bactericidal effect on *Salmonella* itself. For instance, including organic acids (like formic or propionic acid) in poultry feed or water has been shown to reduce *Salmonella* colonisation by creating an unfavourable intestinal pH for the pathogen [30]. Similarly, adding competitive exclusion products (probiotics, prebiotics) to feed can help establish gut microflora that outcompete *Salmonella*. A recent review highlighted that dietary interventions, such as probiotics, organic acids, and phytochemicals, can effectively reduce *Salmonella* loads in poultry when integrated into a comprehensive programme [12,31]. Nutritional strategies are thus a valuable addition to hygiene measures for controlling *Salmonella* before harvest.

Water quality is another vital yet often overlooked aspect. Water lines can harbour *Salmonella* in biofilms that protect bacteria from chlorine or other treatments [12]. Once these biofilms establish in a barn’s water system, they can continually introduce *Salmonella* into the drinking water. Regular testing of poultry drinking water for bacterial contamination is recommended, along with the periodic sanitation of water lines (e.g., flushing lines with approved sanitisers or using acidifiers or low levels of chlorine). Studies show that sanitising water lines with acids or sanitisers reduces biofilm and bacterial loads, thereby likely decreasing flock exposure to pathogens like *Salmonella* [11]. Furthermore, preventing wild birds from accessing poultry drinking water (in range systems) is essential [12]. Together, training, awareness, strict feed hygiene, and proactive water-line management form a necessary layer of pre-harvest control.

### 2.2. Swine Farms

**Figure 4:**
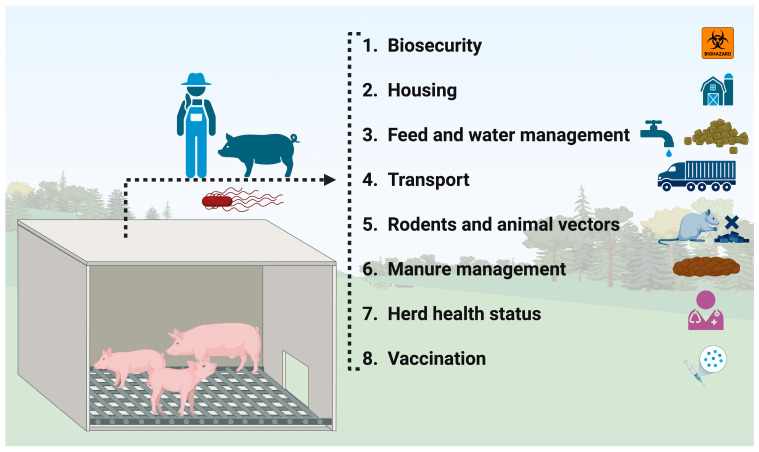
Pig farm management factors involved in salmonellosis control. Created with Biorender.com.

Pigs are another significant reservoir of non-typhoidal *Salmonella*, especially *S. enterica* serovars like Typhimurium, Derby, and Infantis, which commonly colonise swine. In Europe, *Salmonella* in pork has consistently been a challenge—while some countries (e.g., Denmark, Sweden) have longstanding control programmes, others still report moderate to high *Salmonella* prevalence in finishing pigs and pork products. Subclinical infections and the lack of a single solution complicate pre-harvest control in pigs [5]. However, recent studies have highlighted how various farm management factors can significantly influence *Salmonella* levels in pig herds. Here, we review key practices—biosecurity, pig flow and housing, feed and water, antibiotic use, and vaccination—with a focus on findings from EU contexts. Many EU member states have developed biosecurity scoring tools and guides for pig farms, underscoring the importance of biosecurity. The most common management factors involved in reducing salmonellosis control at the farm level are presented in Figure 4.

#### 2.2.1. Biosecurity

Biosecurity in pig farms shares many of the same principles as poultry. Preventing the introduction of *Salmonella* via incoming pigs, feed, or visitors is the first line of defence. Procurement of breeding stock from *Salmonella*-free herds, or testing and quarantining new pigs, is strongly recommended. External biosecurity measures, such as controlled entry for vehicles, people, and equipment, are crucial because *Salmonella* can readily be introduced via livestock markets or transport trucks. Many large pig operations restrict outside visitors and enforce strict entry protocols (showering in, changing clothing/boots) to mitigate this risk. Practices such as sourcing pigs from *Salmonella*-monitored or *Salmonella*-certified-negative herds, quarantining new arrivals, restricting farm access, and thoroughly cleaning transport trucks can significantly reduce the introduction of *Salmonella* [32,33]. This highlights the importance of purchasing replacement pigs exclusively from high-health-status herds and, where feasible, maintaining a closed herd. Farms with strict entry policies for pigs, visitors, and vehicles had markedly lower odds of *Salmonella* infection compared to those with more relaxed entry controls. Sanitation between batches, especially in farrowing and nursery pens, helps break transmission cycles. Notably, one study found that even after cleaning, *Salmonella* can persist in pens, so disinfectant choice and contact time are critical—some farms use heated pressure washing and pen drying to improve efficacy. Flooring design also matters; fully slatted floors that separate pigs from faeces may reduce oral re-infection compared to solid floors with bedding, though they pose other animal welfare trade-offs [15,34,35,36].

Internal biosecurity measures include grouping pigs by age, avoiding cross-contamination between pens, and implementing all-in/all-out batch management, particularly in nursery and finishing units. These practices aim to break transmission cycles within the farm. One widely recommended approach is the all-in/all-out system for pig groups. Under this system, barns (or at least sections or pens) are managed in batches: pigs of the same age enter together, leave together for slaughter, and the facility is thoroughly cleaned and disinfected before the next batch enters. This breaks the continuous presence of animals that could sustain *Salmonella* in the environment [12]. Other internal biosecurity measures include grouping pigs by age, avoiding cross-contamination between pens (e.g., via equipment, boots, and other objects), and controlling vectors on the farm. Although pigs are usually kept indoors, rodent and fly control should not be overlooked, as rodents can move between farms or barn sections carrying *Salmonella*. A recent meta-analysis identified all-in/all-out production as a protective factor (OR ~0.7) on *Salmonella* pig farms, though the results were variable [37].

#### 2.2.2. Housing

The housing system and environment in which pigs are raised can influence *Salmonella* exposure and persistence, with most commercial swine in the EU raised on slatted-floor indoor systems, which allows faeces and waste to fall into a pit away from the animals [38,39]. This can help reduce direct faecal–oral recycling of *Salmonella*. In contrast, pigs on solid floors with bedding, or in outdoor or free-range systems, may have greater contact with manure or soil, increasing the opportunity for oral exposure to *Salmonella*. Comparative studies of indoor versus outdoor and conventional versus organic pig farms have reported inconsistent patterns in *Salmonella* prevalence: some found higher infection levels in outdoor or free-range herds, others detected no significant difference between systems, and some observed higher prevalence in intensive indoor herds [20,40]. Collectively, these mixed findings support the view that housing type (indoor versus outdoor) is less decisive than the quality of farm biosecurity and hygiene, such as cleaning and disinfection practices, control of pig mixing, feed and water management, and rodent exclusion, in determining the *Salmonella* status of pig herds [36].

Within indoor systems, barn design and sanitation are essential. Pens should be arranged to reduce contact between different age groups. Nurseries, growers, and finishers should ideally be located in separate buildings or rooms, with separate airflow, to prevent cross-infection. Ventilation and climate also play a role—high humidity and poor airflow can prolong *Salmonella* survival in the barn environment. Regular removal of manure from pits and effective manure management practices (including storage, composting, or biogas digestion) can help reduce pathogen levels over time. However, these effects are less well-documented in *Salmonella* than in indicator bacteria. Some European farms use manure biogas or treatment systems that are expected to help kill pathogens [41]. This document indicates that manure management and treatment (e.g., anaerobic digestion-biogas) can reduce pathogens and emissions, with techniques including mesophilic/thermophilic digestion, thermal inactivation (pasteurisation/sterilisation), and hygiene processes (e.g., ammonia treatment, composting, drying, acid treatment), often involving strict temperature/pH control to achieve a safe digestate for spreading or reuse, aiming for pathogen reduction levels. A notable factor is farm size and density: large-scale farms with continuous pig throughput can amplify *Salmonella* spread unless very tightly managed. Herds with more pigs or higher stocking density per pen have shown a higher probability of *Salmonella*, likely because of increased animal-to-animal contact and stress [37]. However, large farms can effectively control *Salmonella* by implementing comprehensive measures (as observed in the Danish programme, where even very large herds achieved low prevalence through rigorous monitoring and control). If downsizing is not an option, it is crucial to focus on minimising stressors and improving hygiene. Pigs under stress (from overcrowding, heat stress, transport, or mixing) tend to shed more *Salmonella*, even if they were previously carriers at low levels. Therefore, gentle handling, avoiding sudden mixing of unfamiliar pigs, and providing adequate space can help maintain low *Salmonella* shedding. Another environmental aspect is lairage and transport. Although technically post-farm, these stages feed back into farm management because pigs often become infected with *Salmonella* in markets or slaughterhouse holding pens. *Salmonella* can be acquired during transport or lairage and subsequently reintroduced to the farm if the pigs are returned (e.g., unsold at market). Thus, farm management best practice is that all pigs leaving the farm go to slaughter (no returns), and transport vehicles are cleaned/disinfected before loading at the farm and after use [10].

#### 2.2.3. Feed and Water Management

Diet modifications have demonstrated promise in reducing *Salmonella* in pigs. Acidification of feed or water (e.g., adding formic or lactic acid) has been strongly associated with lower *Salmonella* levels in several studies [15]. Pigs consuming acidified feed or water showed approximately 75% lower odds of *Salmonella* excretion, likely due to direct bactericidal effects in the feed and gut, as well as a shift towards a more acidic, less favourable intestinal environment [15]. Rodent control is again critical on pig farms, as rodents can spread and amplify *Salmonella* by contaminating feed and the environment. Consistent deratisation programmes can significantly decrease *Salmonella* burdens. This highlights the importance of management-focused interventions over production system labels when designing effective strategies to control *Salmonella* in pig production [42].

Feed additives present another promising approach. Various additives have been tested to reduce *Salmonella* in pigs: organic acids (such as formic and fumaric), medium-chain fatty acids (derived from coconut or palm oil), prebiotics (like oligosaccharides that bind *Salmonella*), probiotics (such as *Lactobacillus* for competition), and substances such as essential oils or phytochemicals. In vitro and in vivo studies show that medium-chain fatty acids can exert bactericidal or bacteriostatic effects against *Salmonella* and other enteropathogens while also modulating the gut microbiota in weaned pigs [43,44,45]. Probiotics and synbiotics (combinations of probiotics and prebiotics) are reported to enhance gut health and competitive exclusion, thus reducing *Salmonella* colonisation pressure [1,43,44,45]. No single additive is a complete solution, but each can contribute modestly to control. Significantly, these alternatives are increasingly being adopted as the swine industry seeks to replace prophylactic antibiotics. Interestingly, beyond just hygiene, the physical form and composition of feed markedly influence *Salmonella* in the pig gut. Research over the last decade has shown that feed form (pellet vs. mash) and particle size can alter the risk of *Salmonella* colonisation. Coarsely ground mash feed has been associated with lower *Salmonella* prevalence in finishing pigs compared to finely ground pelleted feed [1]. The likely mechanism is that coarse feed encourages chewing and saliva production, leading to more acidic gastric pH that kills *Salmonella*, whereas finely ground pellets are consumed quickly and result in higher gut pH, which *Salmonella* can survive [1,46,47]. Additionally, fermented liquid feed (FLF) has gained attention: fermenting feed (with lactic acid bacteria) prior to feeding pigs can produce organic acids that reduce *Salmonella* levels in the feed and gut [48].

Water hygiene is also equally critical in pig production for reasons related to vaccination. Pigs consume large volumes of water, and the contamination of drinking systems can rapidly expose entire pens. Although there are fewer studies specifically on water in pigs than in poultry, the basic principles for ensuring safe water still apply [49]. However, research suggests that certain vaccines or competitive exclusion strategies may reduce *Salmonella* shedding in pig herds. For example, studies have demonstrated that vaccinated sows can transfer maternal antibodies that help piglets resist *Salmonella* colonisation early on [15,50]. In Europe, vaccination is considered part of an integrated control approach, but the cost–benefit ratio is carefully evaluated [43,51]. A systematic review of pre-harvest interventions found that feed-based treatments, such as probiotics and organic acids, alongside vaccination, show promise for *Salmonella* control. However, evidence for vaccination under commercial field conditions remains limited in demonstrating large-scale, reproducible reductions in prevalence [43].

Farm workers can both acquire *Salmonella* from pigs and transmit it between groups of animals. Staff training in hygiene, such as handwashing, boot disinfection, and the use of dedicated tools for each barn, helps prevent the spread of the disease [52]. Ideally, employees should work sequentially from younger to older pigs, as older finishing pigs are more likely to be carriers, or use separate personnel for different units. Emphasising personal hygiene (e.g., washing hands and changing coveralls) when moving between pig groups has been highlighted in biosecurity guidelines [53]. Overall, effective management practices on swine farms include: strict biosecurity (especially rodent control, controlled pig introductions), feed and water acidification, all-in/all-out pig flow, and diligent sanitation [15]. From a One Health perspective, these reductions contribute to lower environmental contamination and reduced transmission throughout the pig production chain.

### 2.3. Cattle and Dairy Farms

**Figure 5:**
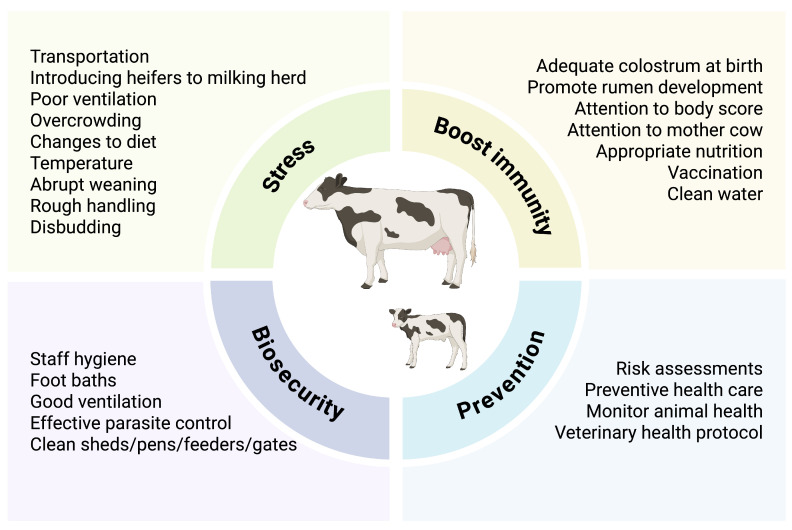
Factors responsible for increased susceptibility to salmonellosis in cattle. Created with Biorender.com.

Although infections are often asymptomatic in farm animals, a range of factors, including stress, immunity, biosecurity, and preventive measures (Figure 5), influence the management of *Salmonella* infection in cattle, which can cause sporadic clinical disease (e.g., calf scours from *S*. Dublin) but more commonly results in asymptomatic carriers that shed in manure.

Beef cattle, especially in feedlots, and dairy cattle (which can contaminate milk or the farm environment) are essential to manage for *Salmonella,* given the downstream risks to meat and dairy products [54]. Beef feedlots can have high *Salmonella* carriage in animals destined for slaughter, potentially contaminating meat (particularly ground beef via lymph nodes) [55]. Recently, there has been increasing focus on controlling *Salmonella* at the farm level as part of a “One Health” strategy, rather than relying solely on abattoir interventions [10,56].

#### 2.3.1. Biosecurity and Herd Management

The main ways *Salmonella* enters a farm are through infected animals, contaminated feed or water, and vectors such as wildlife, rodents, birds, and insects [10]. Among these, the introduction of a carrier animal is considered the greatest risk. Unlike closed poultry flocks, cattle herds often acquire new stock (breeding heifers, bulls, or calves), which may originate from external sources with unknown *Salmonella* status. Research has long demonstrated that purchasing and replacing animals are associated with *Salmonella*-positive herds [10]. A new cow may appear healthy but can intermittently shed *Salmonella*, contaminating her environment. Long-term studies have shown that shedding can persist for many months after infection, and in some *S.* Dublin-infected herds for even more extended periods (up to a year), highlighting how a single carrier can sustain contamination [57]. *S.* Dublin, a bovine-adapted serovar, is well-known for causing chronic, often subclinical infections; animals that appear clinically recovered may continue to shed the pathogen intermittently and silently infect herd mates over extended periods [10,57]. Therefore, maintaining a closed herd or ensuring careful control of animal introductions is essential. Farms should source replacements from *Salmonella*-free herds or suppliers with testing programmes. At a minimum, new arrivals should be quarantined and tested (e.g., via faecal culture or serology) before mingling with the existing herd [10].

Cattle operations, whether feedlots or dairies, benefit from many of the same biosecurity principles [54]. Preventing the introduction of *Salmonella* into a naive herd involves sourcing animals from known *Salmonella*-negative herds where possible, using serology for *S.* Dublin in dairy replacements, and avoiding the commingling of cattle from multiple sources (or, if unavoidable, implementing strict quarantine and isolation) [10]. In feedlots, where commingling is inherent, emphasis is placed on external biosecurity at the facility: designated “clean” routes for feed delivery, restricted visitor access, and the use of clean trucks for animal transportation. Outbreak investigations highlight the significance of external biosecurity: for example, a multi-state outbreak of *S.* Heidelberg in the U.S. was traced to dairy bull calves sold through livestock markets, illustrating how animal trading can widely disseminate *Salmonella* [58].

Practical recommendations include controlled farm entrances, such as parking areas away from barns and transitional sanitation stations, to prevent contamination from external vehicles or people from tracking in [59]. Some larger dairies now require milk tankers and feed trucks to disinfect their tyres or to use drive-over antimicrobial pads upon entry. Farm equipment (e.g., livestock trailers, feed trucks) and personnel can carry *Salmonella* between farms if not appropriately managed; cleaning and disinfection of livestock trailers after use are increasingly adopted, and some EU dairy farms have implemented truck disinfection protocols similar to those used in the swine and poultry sectors [60]. Visitors and veterinarians should wear clean boots and clothing; footbaths and handwashing on entry and exit help reduce pathogen transmission [10]. Rodents and wild birds can also spread *Salmonella* on cattle farms. For example, small birds may contaminate feed bunks, and rodents can move between feed stores and cow areas. Effective rodent control methods, such as baiting and barn proofing, along with measures like covering feed and water sources, can help reduce this risk. Filth flies have been linked to the spread of *Salmonella* in feedlots during warm months, so manure management and fly control are also critical [61].

#### 2.3.2. Hygiene and Sanitation

Maintaining a clean and dry environment for cattle helps reduce *Salmonella* persistence. On dairy farms, regular manure removal and proper storage, such as composting instead of spreading raw manure on pastures, can interrupt transmission cycles. *S.* Dublin can spread in calf huts via contaminated bedding; therefore, cleaning and disinfecting calf pens between groups is essential [62,63]. Cleaning and disinfecting calf pens should follow standard dairy biosecurity guidelines, with the complete removal of bedding and manure, washing with detergent, thorough rinsing, and application of an approved disinfectant at the manufacturer-recommended concentration and contact time, followed by a drying period before new calves are introduced. Individual pens or hutches should be fully cleaned and disinfected between calves, and heavily soiled bedding removed at least daily, with full bedding replacement at regular intervals (e.g., weekly), according to stocking density and climatic conditions [64]. Water sources must be protected; cattle should not access stagnant water that could be contaminated by wild birds or other animals. For grazing beef cattle, preventing contact with surface water that receives runoff from other farms decreases the risk of *Salmonella*. Feed hygiene is also vital; using *Salmonella*-free feed is crucial, as animal-origin ingredients or pests can contaminate feed. The U.S. FDA’s Food Safety Modernisation Act introduced preventive controls for animal feeds, including safeguards against *Salmonella* for most commercial feed producers [8,65]. On the farm, proper feed storage, such as sealed bins and rodent-proof containers, along with correct handling to avoid cross-contamination between raw ingredients and feed bunkers, is a standard good practice.

Strict hygiene in calving areas and calf-rearing facilities is essential. Newborn calves often acquire *Salmonella* from the environment or by ingesting contaminated colostrum. Management guidelines advise that cows known to be *Salmonella*-positive (by faecal testing or herd history) should calve in separate, isolated maternity pens away from uninfected cows [10]. Calving pens should be kept meticulously clean and regularly disinfected, as *Salmonella* can heavily contaminate bedding during outbreaks. Placentas and heavily soiled bedding should be promptly removed and disposed of, and clean, dry bedding should be provided for each calving. After calving, colostrum management is essential: feed calves only colostrum from *Salmonella*-negative cows, or pasteurise colostrum if the status is uncertain [10]. Similarly, milk (or milk replacer) given to calves should be free from *Salmonella*—pasteurisation of waste milk is one strategy to eliminate pathogens. In endemic herds, feeding only milk replacer (under appropriate veterinary guidance) can minimise calf exposure to *Salmonella* shed in cows’ milk. Field experience and observational studies suggest that these practices can significantly reduce calf scours and *Salmonella* outbreaks in calves [10].

A comprehensive cleaning and disinfection (C&D) programme can break that cycle. For example, during an outbreak of *S.* Bredeney on an Italian dairy farm, control measures included removing all animals from the pen, hot-water pressure washing with detergents, disinfecting with phenolic compounds, and leaving the pens empty to dry; this strategy helped eliminate the pathogen after repeated prior reinfections [66]. High-pressure hosing while cattle are present is discouraged because it can aerosolise and spread bacteria [67]. In barns, using foam or low-pressure sprayers with detergents can gently loosen biofilms and organic dirt without causing significant aerosolisation [68,69]. After cleaning, exposing surfaces to sunlight and allowing them to dry helps inactivate *Salmonella*, which is less tolerant of UV and desiccation than of cool, wet, shaded conditions [70,71,72]. In outdoor corrals or paddocks, practical measures include periodically scraping and removing the upper layer of manure and mud and avoiding overstocking so that ground surfaces can dry and receive sunlight. These steps are biologically plausible ways to reduce environmental load, although their quantitative impact on *Salmonella* in cattle housing has not been robustly quantified [10,72].

Another aspect is manure management during in-house feeding or outdoor grazing. Cattle can become infected with *Salmonella* through contamination of their feed or environment with manure. If properly composted to reach and sustain high internal temperatures (e.g., ≥55–65 °C for an adequate period), manure or used bedding can considerably decrease or eliminate *Salmonella* spp., making composted material much safer for field application than raw manure [73,74]. In yards and pastures, adequate drainage and avoiding persistent standing water or heavily muddied areas are vital, as such wet, shaded zones support the long-term survival of *Salmonella* and can promote contamination (including via wild birds and rodents) [74,75].

#### 2.3.3. Animal Handling, Stress, and Stocking Density

Stress is a recognised factor that can increase *Salmonella* shedding in cattle. Events such as transport, overcrowding, calving, or sudden diet changes can trigger higher faecal shedding. Therefore, low-stress handling techniques (gentle handling of cattle, avoidance of electric prods, and provision of adequate space) are beneficial not only for animal welfare but also for food safety. In feedlots, cattle are often given a diet transition period on arrival and before slaughter to stabilise the gut. High stocking densities, continuous animal movement in and out, and frequent mixing of unfamiliar cattle (as in many feedlots) heighten the challenge. While reducing herd size may not be feasible, farms can mitigate risk by subdividing large groups into smaller units, improving ventilation, and practising batch management for young stock. All-in/all-out rearing of calves and heifers (raising groups of similar age together, with no new additions until that group departs) has been recommended to decrease *Salmonella* transmission to susceptible young animals. Young calves are particularly vulnerable to salmonellosis; therefore, maintaining separate age groups and avoiding the introduction of older carrier animals into calf areas provides additional protection [76].

#### 2.3.4. Feed and Water Management

Recent monitoring of livestock feed has revealed *Salmonella* contamination in about 7% of feed component samples, with higher rates found in blended feeds, animal protein meals, and oilseed meals [77]. Dairy and beef cattle are usually fed total mixed rations (TMR) that include silage, hay, grains, and various by-products. Silage and moist by-products can promote bacterial growth if ensiling or storage is inadequate. Reviews of silage microbiology indicate that poorly fermented or aerobically spoiled silages can harbour foodborne pathogens, including *Salmonella*, whereas rapid lactic acid fermentation, resulting in sustained pH decrease and anaerobic conditions, greatly limits their survival [78,79,80]. Practically, visibly spoiled or mouldy silage at the feed-out face should be discarded. Feed storage should also prevent contamination by pests and wildlife by keeping bins covered, using bird-proof lids, and promptly removing spilt feed to deter rodents and wild birds.

Alongside hygiene measures, there is growing interest in nutritional and pre-harvest interventions to reduce *Salmonella* in cattle. Some feedlots administer direct-fed microbials (probiotics), and experimental studies have evaluated short-term feeding strategies, such as sodium chlorate supplementation or vaccination, to reduce pathogen loads before slaughter [6]. To date, such chemotherapeutic approaches (e.g., sodium chlorate) remain experimental and are not routinely used in commercial practice, but they demonstrate the range of potential tools. From a nutritional perspective, research is exploring how diet influences *Salmonella* in cattle. Unlike pigs, cattle have a rumen whose acidic, volatile fatty acid-rich (VFA) environment can inactivate many ingested *Salmonella*, at least under typical forage-based feeding conditions [55]. Some authors have hypothesised that extremely high-starch diets, which predispose to subacute ruminal acidosis and damage to the rumen epithelium, could facilitate the translocation of enteric bacteria, including *Salmonella*, across the rumen wall; however, direct evidence for this pathway remains limited and mostly inferential [55]. Conversely, certain feed additives may help reduce gut carriage. Direct-fed microbials (DFMs), such as Lactobacillus or Propionibacterium cultures, have shown promise in reducing faecal *Salmonella* shedding or mitigating health and performance impacts in *Salmonella*-challenged calves in some experimental studies, although field results are variable [55,81,82]. Dietary inclusion of yeast or yeast-derived supplements (e.g., *S. cerevisiae* products, β-glucans, mannans) has also been associated with improved gut health and reduced enteric pathogen burdens in livestock; however, for cattle specifically, outcomes remain inconsistent and depend on factors such as additive type, dose, animal age, and management [83,84].

Water for cattle is often sourced from wells or municipal supplies, which are generally low-risk for *Salmonella* contamination. However, water troughs can become contaminated with cow saliva and manure. Regular cleaning of troughs is recommended; some farms also sanitise water (e.g., low-dose chlorine), especially in calf barns. Protecting water sources from birds (e.g., installing physical deterrents on troughs in outdoor pastures) and preventing access to stagnant ponds or puddles that may be contaminated by wild birds or wildlife are sensible measures [85]. Good pasture management (draining low spots, fencing off natural ponds, or treating the water) can help reduce these risks.

#### 2.3.5. Pest and Wildlife Control

Cattle facilities must deal with rodents and wild birds. Birds such as starlings and pigeons that feed on cattle feed can spread Salmonella through their droppings, making it necessary for large feedyards to regularly implement bird-deterrence programmes. Rodent control in and around feed storage and barn areas is equally important, as rodents can shed Salmonella acquired from other farms or the environment [10]. In pasture-based systems, cattle may be exposed to Salmonella from wildlife (for example, deer or feral pigs) that access grazing areas and deposit contaminated faeces or access water and forage. This route of exposure is more challenging to control than many indoor risks, so farms focus on maintaining adequate fencing, restricting wildlife access, and minimising attractants (e.g., spilt feed, water ponds) that draw wildlife near herds [10,86].

#### 2.3.6. Training and Protocols

On dairy farms, workers must carefully follow hygiene practices when moving between groups of animals. A common best practice is to attend to young calves first, then older animals, to prevent the transmission of pathogens from adults (who may be carriers) to vulnerable calves [10]. Wearing gloves when assisting births or handling sick animals and washing hands afterwards helps prevent the spread of manure-borne infections. Hygiene during milking is vital: while pasteurisation kills *Salmonella* in milk, pre-harvest measures such as keeping udders clean and sanitising milking equipment help reduce raw milk contamination. Therefore, standard operating procedures for cleaning milking parlours and equipment (CIP systems), and for proper pasteuriser operation in on-farm dairy processing, are essential management practices. Training staff in these protocols and fostering a strong food safety culture (even on the farm) support consistent compliance [10].

## 3. *Salmonella* and AI in Livestock Farm Management

**Figure 6:**
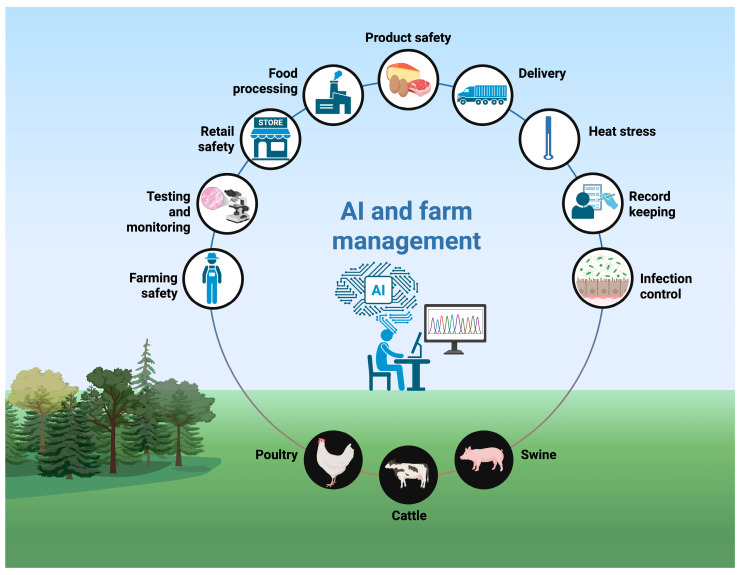
Farm management tools with potential impact on salmonellosis control. Created with Biorender.com.

Taken together, the traditional management practices described in this section—spanning biosecurity, hygiene, vaccination, feed and water management, and antimicrobial use—form the current backbone of farm-level *Salmonella* control across poultry, swine, and cattle systems. However, their effectiveness often depends on timely risk detection, consistent implementation across diverse production environments, and the ability to integrate large volumes of heterogeneous information (e.g., environmental monitoring, production records, animal health data). These are precisely the areas where artificial intelligence and data-driven tools can provide added value. In the following section, we discuss how AI-based approaches can support and enhance these established strategies by improving surveillance, risk prediction, and decision-making at the farm level. Although analytical methods are not, per se, farm management interventions, improved analytical performance can have a decisive impact on management decisions at the primary production level. More sensitive and rapid detection of *Salmonella* in animals, feed, water, and the farm environment enables the earlier identification of infections and contaminated sources, thereby enabling the timely implementation or intensification of hygiene, biosecurity, and movement control measures. In addition, reliable analytical tools support the longitudinal monitoring of intervention effectiveness, allowing farm managers to adapt or prioritise control measures based on objective evidence rather than clinical signs alone. In this way, advances in analytical performance indirectly but substantially influence farm management practices aimed at reducing *Salmonella* risk. Artificial intelligence has been widely used in the management of poultry, dairy, and pig farms since the term was first introduced in the ‘50s [87]. Over the past 70 years, every advancement in the field has focused on improving farm management, particularly by reducing manual labour, minimising environmental impact, and decreasing reliance on extensive economic resources [88]. Combining traditional biosecurity measures with modern AI technologies to reduce the incidence of harmful pathogens is challenging, as the terminology for crosslinking remains scarce. In Figure 6, we present the likely management tools available at the farm level for the control and monitoring of salmonellosis.

**Figure 7:**
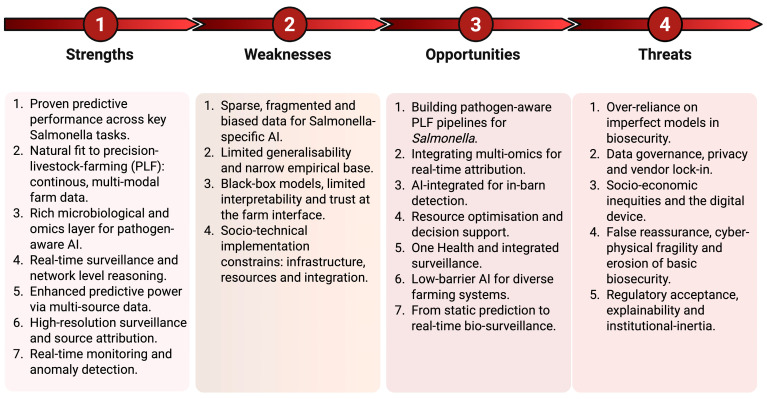
SWOT analysis of AI’s impact on *Salmonella* management at the farm level. Created with Biorender.com.

This lack of information may also reflect perceptions of AI in the field, as it remains an evolving area with limited understanding of its benefits. Therefore, we conducted a SWOT analysis as a starting point (Figure 7) and further discussed the strengths, weaknesses, opportunities, and threats in detail.

### 3.1. Strengths

***Proven predictive performance across key Salmonella tasks*****.** Non-destructive optical and spectroscopic methods, combined with machine learning (ML), have demonstrated promising predictive performance across key *Salmonella*-related tasks, including the detection of contamination and associated microbial changes in poultry products. FTIR spectroscopy, in conjunction with supervised learning, has enabled the rapid microbial quality assessment of chicken liver, whether inoculated with *Salmonella* or not, demonstrating that spectral fingerprints and ML can reliably identify subtle shifts in microbial load and composition [89,90]. Broader spectroscopic research documents successful applications of hyperspectral and vibrational spectroscopies, again paired with ML, for detecting foodborne pathogens, including *Salmonella,* on meat surfaces and within complex matrices [89,90,91,92].

***Natural fit for Precision Livestock Farming (PLF): generates continuous, multi-modal farm data.*** Since *Salmonella* ecology is closely linked to animal density, stress, litter, manure conditions, and microclimate, these PLF variables serve as a natural foundation for AI-based risk modelling. Early research in antibiotic-free, pastured poultry systems has already utilised ensemble ML to link management variables, soil and faecal physicochemistry, and meteorological factors with *Salmonella* prevalence, identifying combinations of flock size, years of farm use, trace elements, and soil electrolytes that influence the risk of contamination [93,94]. Other studies have used environmental and management variables to forecast pathogen presence in agricultural water and to rank risk factors in pre-harvest food safety contexts [93,94]. These case studies show that PLF infrastructure designed for welfare and productivity can be adapted as precise bio-surveillance tools for *Salmonella*, especially when regular microbiological sampling is combined with sensor data.

***Rich microbiological and omics data layers enable pathogen-aware AI**.* The essential microbiological and omics layers relevant to *Salmonella* are already highly “AI-ready”. Genome-based ML models predict AMR phenotypes and even MICs directly from whole genome sequencing (WGS) data, including isolates from chicken meat, using pan-genome and pan-resistome features with high predictive accuracy [95,96]. FTIR workflows supplemented with multivariate ML provide rapid serogroup/serovar discrimination with very high concordance to conventional serotyping [90,97]. At the community level, ML applied to metagenomic and microbiome datasets infers resistome structure and pathogen risk across intensive and pastured poultry systems when microbiome features are combined with farm-management and environmental metadata [98,99,100]. Overall, these layers, WGS, spectral typing, and microbiomes, demonstrate that AI can leverage both isolate-level and community-level signatures, offering a strategic advantage for developing *Salmonella*-aware risk models along poultry, cattle, and swine value chains as training datasets expand and strengthen outbreak detection, source attribution, and situational awareness for *Salmonella* and other enteric pathogens.

***Maturing architectures for real-time surveillance and network-level reasoning.*** Beyond individual farms, AI is now used to model *Salmonella* signals at regional, pig, broiler, and supply-chain levels. In north-western Italy, tree-based ML models trained on integrated food-surveillance data and municipal human salmonellosis incidence (2015–2019) achieved strong predictive performance (RF/GB: R^2^ ≈ 0.55, MAPE ≈ 7.5%), supporting their use as an early-warning tool for spatial hotspots and targeted interventions [101]. Recent “farm-to-fork” AI reviews highlight that these architectures are increasingly capable of combining multi-source inputs, sensor streams, laboratory and inspection data, and public health surveillance data into near-real-time risk dashboards for foodborne hazards, including bacterial pathogens [94,102,103,104]. Collectively, these developments constitute a clear strength from a SWOT perspective: core AI architectures for multi-scale, network-aware bio-surveillance already exist and can be tailored for *Salmonella*-focused risk assessment across farms, processing plants, and downstream human cases.

***Enhanced predictive power through multi-source data*****.** AI models can combine weather, geographic, farm-management, and genomic data to forecast *Salmonella* risk with sensitivity and specificity comparable to, or in some cases exceeding, those of traditional methods. For instance, method-focused reviews describe ML models for foodborne pathogens that utilise environmental features to accurately predict *Salmonella* levels on farms [105]. In swine, a predictive regression model was developed using pig serology, pen-positivity, and farm biosecurity to forecast *Salmonella* shedding at slaughter (AUC ≈ 0.76) [106]. These examples illustrate AI’s ability to leverage diverse datasets (farm surveys, serological tests, on-farm monitoring) to identify high-risk flocks or herds before slaughter.

***High-resolution surveillance and source attribution*****.** More broadly, deep-learning models trained on resistome profiles and metadata can predict antimicrobial resistance (AMR) phenotypes and help prioritise interventions in multi-serovar settings [98]. Network-based analyses of large genomic surveillance datasets revealed relationships between specific AMR genes, plasmids, and lineages, informing control strategies at the farm and regional levels. Using 16S rRNA-based microbiome data from faeces and soil of pastured poultry flocks, ML algorithms can predict the presence of foodborne pathogens, including *Salmonella*, and map their associations with putative probiotic taxa [107]. At the genomic level, ML was applied to whole-genome sequences of *Salmonella typhimurium* to improve host (animal reservoir) attribution, demonstrating that WGS+ML can link human cases back to specific animal production sectors with higher resolution than traditional methods [108,109]. Finally, hierarchical models for *S. enteritidis* demonstrate how genomic and epidemiological data can be integrated to elucidate fine-scale transmission patterns among farms, flocks, and human cases [110].

***Real-time monitoring and anomaly detection.*** AI excels at processing continuous sensor inputs for early warning. Systems that combine AI with IoT sensors in barns—monitoring temperature, humidity, manure properties, and animal behaviour—can detect deviations from expected patterns that indicate increased *Salmonella* risk and trigger investigations or targeted sanitation [111]. As highlighted in broader reviews, ML models applied to sensor networks can continuously monitor for pathogens (including *Salmonella*) and environmental drivers, complementing traditional spot sampling [105]. In practice, this could mean the automated detection of faecal contamination on poultry lines or behavioural changes in animals (via computer vision) that precede *Salmonella* outbreaks. Machine-learning frameworks are also beginning to shorten detection times in outbreak contexts: when *Salmonella* risk models are integrated with weather, hydrological, and clinical data, they can forecast outbreak patterns and support intensified sampling or public health interventions before case counts peak [101].

### 3.2. Weaknesses

***Sparse, fragmented, and biased data exist for Salmonella-specific AI.*** At the farm level, genuinely *Salmonella*-labelled datasets remain limited. A 2025 survey of open-access image and video datasets for computer vision in precision poultry farming identified 20 datasets, primarily focused on behaviour monitoring, health status identification, live performance prediction, product quality inspection, and trait recognition. However, none of these datasets longitudinally pair PLF video streams with microbiologically confirmed *Salmonella* status [92]. One of the few disease-related resources is a faecal-image dataset used for mobile poultry health assessment, comprising four classes (healthy, coccidiosis, Newcastle disease, *Salmonella*), but is restricted to single-modality images and does not incorporate barn-level PLF data or laboratory WGS results [92]. Beyond their scarcity, available data are often biased and poorly documented. Large-scale analyses in agriculture show that farmers may omit key production details, selectively report performance, and be hesitant to share sensitive information, resulting in non-representative samples [112]. Management practices (e.g., cleaning frequency, feed formulation, pasture rotation) are often recorded as coarse categorical variables or as self-reported survey responses, which are susceptible to recall and social desirability biases. High-resolution WGS studies on dairy farms similarly highlight that even dense genomic data are challenging to interpret without comprehensive, standardised on-farm metadata such as housing, feed, vaccination, and movement [113]. These issues limit ML models’ capacity to learn robust, transferable patterns and complicate meta-analyses across studies; however, there is a need to coordinate efforts in data standardisation and harmonisation, as well as provide incentives for sharing high-quality data.

***Limited generalisability and a narrow empirical base.*** The empirical foundation for farm-level *Salmonella* models is especially limited. Recent research on pastured poultry involved 11 farms in a single southeastern state in the U.S. [93,107,114], while key dairy studies on management and multidrug-resistant *Salmonella* used data from six Californian herds [115]. Even the well-cited pig-shedding model, which achieved an AUC of approximately 0.76 and a sensitivity of approximately 72%, remains a proof-of-concept developed within a specific production context and has uncertain performance in other regions or husbandry systems [106]. Furthermore, nearly all available studies only report technical metrics (AUC, F1-score, accuracy) and variable-importance rankings; none provide robust evidence that implementing the AI model in practice will reduce *Salmonella* prevalence, carcass or milk contamination, or human disease burden. This combination of limited geographic and system diversity, modest external validation, and the absence of outcome-level evidence indicates a substantial weakness in the current evidence base.

***Black-box models, with limited interpretability and trust at the farm interface, pose a significant challenge.*** Black-box models with limited interpretability pose a major challenge for on-farm adoption of AI tools. For *Salmonella* biosecurity, this opacity is a critical weakness. Unless AI-derived risk scores can be broken down into biologically plausible factors, such as litter moisture, ventilation patterns, stocking density, feed modifications, or antimicrobial-use decisions, farmers and veterinarians are likely to resist recommendations they cannot reconcile with field observations, even when model performance appears strong on held-out datasets. Several studies on *Salmonella* and farm biosecurity deliberately utilise interpretable machine learning. For example, gradient-boosted trees with SHAP values can be used to analyse how dairy management practices influence multidrug-resistant *Salmonella* and commensal carriage in cull cows. Interpretable ensemble models (MrIML-biosecurity) with variable importance and partial-dependence analyses help visualise how individual biosecurity practices impact PRRSV outbreak risk in swine herds [115,116], but many PLF and microbiome models remain essentially black boxes for end-users. Bridging this interpretability gap, through XAI methods, co-designed user interfaces, and transparent communication of model uncertainty, remains an unresolved weakness for farm-level deployment and regulatory acceptance.

***Socio-technical implementation constraints include infrastructure, resources, and integration.*** Despite rapid progress in PLF and agri-AI research, several reviews conclude that AI-enabled sensor systems for livestock health and performance remain largely at the development or pilot stage, with limited evidence of robust, real-time decision support across diverse commercial farms [117]. Infrastructure and resource gaps also influence who can realistically deploy AI. Advanced tools require computational resources, connectivity, and communication that many farms lack, especially smaller or rural operations. Deploying ML dashboards or real-time sensors typically requires reliable Internet access, cloud connectivity, and, in some cases, on-site edge computing hardware. In extensive systems (e.g., transhumant cattle, outdoor pigs) and in many low- and middle-income countries, these conditions are often missing. Reviews of PLF and wearable-sensor systems highlight the significant initial investment needed for hardware, maintenance, and data integration in commercial herds [104,118]. For smallholders, such costs may be prohibitive, raising concerns that AI-based *Salmonella* management tools could disproportionately benefit large, technologically advanced farms while offering limited access to smaller farms.

### 3.3. Opportunities

***Developing pathogen-aware PLF pipelines for Salmonella.*** Recent research indicates that management variables, environmental conditions, and microbiological outcomes can already be linked through ML, offering a blueprint for constructing pathogen-aware PLF pipelines. In antibiotic-free, pastured poultry systems in the southeastern U.S., ensemble models combining deep learning and traditional ML utilised farm-practice variables, along with soil and faecal chemistry, to predict *Salmonella* prevalence and ranked key risk factors such as soil electrolytes, trace elements, flock size, and years of farm utilisation [93,94]. Related work demonstrates how ML can connect management practices and physicochemical variables to performance and barn microclimate in broiler houses, emphasising the feasibility of continuously integrating environmental data and production outcomes through IoT-assisted models [119].

***Integrating multi-omics for real-time attribution and ecological risk profiles.*** AI also offers an opportunity to combine various microbiological and omics data layers into dynamic, clone-aware risk profiles. As sequencing costs decrease, farms or integrators could increasingly implement routine sequencing of *Salmonella* isolates (or, in some cases, shotgun DNA from litter or boot swabs) and perform ML-based analyses on these data. The approach indicates that predictive features, such as virulence genes, can be used to identify isolates associated with specific disease outcomes [120]. Meanwhile, metagenomics-enabled surveillance frameworks demonstrate how deep learning can continuously monitor microbial communities and track emerging threats in complex environments [121]. One key opportunity is to develop proactive, real-time on-farm surveillance networks. By integrating AI with ubiquitous sensors (environmental monitors, wearable animal biosensors, etc.), farmers could identify anomalies and infection risks in real-time and intervene promptly. For example, a mobile robot integrated with IoT-based sensing has been proposed to monitor litter and flag conditions conducive to *Salmonella* proliferation, thereby triggering targeted sanitation or other preventive actions before contamination becomes established [122].

***AI-integrated biosensors and nano-biosensors are used for in-barn detection**.* ML and DL models are increasingly applied to interpret complex biosensor signals—such as impedance spectra, SERS fingerprints, and fluorescence patterns—enhancing detection limits and robustness in complex food matrices. Recent reviews on AI-assisted biosensors for foodborne pathogens indicate that ML applied to electrochemical impedance, SERS, and fluorescence data often achieves high (>90%, and in some studies >95%) classification accuracies for bacterial detection tasks when proper feature extraction and modelling workflows are employed [123,124,125,126]. Meanwhile, work on nanobiosensors for plant health demonstrates that platforms based on carbon nanotubes, graphene, and quantum dots can detect volatile or molecular signatures of plant–parasitic nematodes and related pests, with AI models inferring pest presence and load from noisy, multidimensional sensor output [123,124,127,128].

***Precision interventions, resource optimisation, and decision support***: A good balance between innovation and scientific caution, with a clear link to automation and operational management. Such targeted interventions could reduce unnecessary interventions while enhancing control efficiency, with potential downstream benefits for food safety and public health. AI-driven risk models enable precision biosecurity, allowing interventions to be targeted to high-risk animals, pens, or farms rather than applied uniformly. When a model identifies specific flocks or pens as high-risk, measures such as vaccination, targeted use of probiotic feed additives, or intensified cleaning can be implemented only where necessary, thereby optimising the use of labour and resources. Although not yet proven in large-scale field trials for *Salmonella*, this conceptually expands AI’s role from mere surveillance to active control [129]. Combining AI predictions with farm-management software or robotics could further facilitate timely and standardised responses to emerging biosecurity threats: for instance, mobile robots designed for litter sanitation might be directed to high-risk housing areas, while environmental control systems could automatically adjust temperature, ventilation, and litter-drying as the risk model indicates. Reviews of AI in food safety and precision livestock farming explicitly discuss such “sense–think–act” paradigms, in which AI outputs are connected to actuators rather than used solely for passive surveillance [130,131].

***One Health and integrated surveillance.*** A One Health perspective creates synergy by explicitly linking farm-level AI tools with human health, environmental, and cross-border information flows. The GraphSAGE-based dairy supply-chain model and the Italian ML early-warning system already demonstrate that AI can connect food-chain nodes and public health data into actionable, large-scale risk maps [101,102]. Digital surveillance platforms further argue that integrating food-safety data streams with clinical and electronic health record infrastructures can strengthen outbreak detection and attribution for *Salmonella* and other enteric pathogens [101,132,133]. Moving forward, farm-level AI alerts (e.g., detecting rising *Salmonella* signals in a region’s pig or broiler farms) could feed national monitoring systems in near real-time, enabling regulators to anticipate foodborne outbreaks and implement risk-based surveillance strategies. AI could also support evidence-informed policy instruments by prioritising inspection frequencies, defining targeted sampling strategies, or supporting export certification by identifying sectors or regions with persistently low risk [134,135]. Furthermore, AI results should be treated as decision-making tools rather than automated triggers, and they should be validated by experts before any regulatory action is taken.

***Inclusive, low-barrier AI for diverse farming systems*****.** A recent systematic review on AI for illiterate farmers highlights that while most current tools are not yet deliberately designed for low-literate users, smartphone-based advisory systems with locally adapted, highly simplified interfaces (icons, local languages, minimal text) and mobile/edge deployment are the most promising pathway to inclusion [136]. This is exemplified by the Urdu-language IoAT smart-farming platform developed for low-literate Pakistani farmers, which combines low-cost sensors with a bilingual Android app designed around simple, culturally appropriate interactions [137]. For *Salmonella* management, these approaches create a clear opportunity to deploy lightweight, interpretable models (e.g., distilled trees or rule-based systems) directly on barn-level devices fed by PLF and biosensor streams, and to present outputs as simple traffic-light or voice prompts (“litter too wet—increase ventilation; schedule boot-swab sampling”) rather than abstract risk scores. When executed effectively, such systems could extend AI-enabled biosecurity beyond vertically integrated corporations to smallholders, backyard flocks, and emerging dairy/pig sectors, where baseline *Salmonella* control is often weakest.

***From static prediction to real-time bio-surveillance.*** Many existing *Salmonella*-related ML models operate as offline prediction tools, trained on historical datasets and used for scenario analysis. A short-term opportunity is to transform these frameworks into real-time bio-surveillance systems. For instance, random forest and deep ensemble models developed for pasture poultry could be combined with on-farm weather stations, soil-moisture sensors, and simple sampling devices to produce dynamic “*Salmonella* weather” alerts that identify periods of increased environmental risk and trigger temporary enhancements in hygiene, pasture management, or egg-collection protocols [93,114].

### 3.4. Threats

***Over-reliance on flawed models and deterioration of fundamental biosecurity measures pose significant risks.*** Blind trust in AI outputs is a major danger. All models have limitations: a system trained on large poultry houses may fail to identify new risk factors in free-range or backyard flocks, or in production systems with different breeds, climates, or management practices. If farmers or regulators rely mainly on AI recommendations, such as adjusting stocking densities, changing culling patterns, or relaxing surveillance, without expert oversight, they could misclassify low-risk farms as high-risk, wasting resources and damaging trust, or worse, miss emerging outbreaks. Reviews on AI in food safety consistently highlight that AI tools are “only as strong as the data that feed them” and must be incorporated as advisory components within broader HACCP and One Health frameworks rather than replacing hygiene and basic biosecurity [105,138,139,140].

***Data governance, privacy, and vendor lock-in are significant concerns.*** A related threat is vendor lock-in. As agricultural sectors digitalise, a small number of proprietary platforms and sensor ecosystems are beginning to dominate data collection and analysis [141,142,143,144]. Once a farm invests in a vertically integrated hardware–software system, switching providers can be costly or technically challenging, especially if data formats and models are not interoperable. If the vendor’s algorithms are not transparent, independently validated, or aligned with public-health objectives, farms may become reliant on systems whose actual effectiveness for *Salmonella* control is unclear, and whose incentives are more strongly driven by data monetisation than by reducing contamination [143].

***Socio-economic inequities and the digital divide.*** There is a clear risk that AI will deepen the divide between technology-rich and technology-poor producers. Wealthier, integrated operations are better equipped to invest in sensors, connectivity, and data-science capacity. They can, therefore, leverage AI tools to improve efficiency and risk management, whereas smallholders and backyard producers face challenges with upfront costs, unreliable connectivity, and limited technical support. As previously noted, even encouraging farmers to participate in digital platforms can be difficult, and the benefits of data-driven tools often accrue unevenly across producers [144].

***False reassurance, cyber-physical fragility, and erosion of basic biosecurity.*** AI-based *Salmonella* tools are susceptible to false reassurance if farmers and regulators over-rely on risk scores and overlook bias, data gaps, and limited external validity. Recent AI–food safety reviews and policy reports emphasise that such models should serve as advisory layers within Hazard Analysis and Critical Control Points HACCP and One Health frameworks, rather than replacing hygiene and biosecurity fundamentals [129,138,145,146]. If operators believe that an apparently “green” dashboard confirms low risk, they may neglect routine inspection, environmental sampling, or critical thinking, allowing an outbreak to smoulder undetected when a novel strain emerges or sensors malfunction.

At the same time, increasing reliance on connected PLF and cloud infrastructures introduces cyber-physical vulnerabilities. Ransomware, data tampering, or IoT exploits in smart barns could skew inputs or disable AI monitoring during critical periods, directly endangering food safety [147,148]. In a tightly integrated AI ecosystem, a successful attack on gateways or cloud services might simultaneously disrupt data flows for multiple barns or integrators, impairing model performance and obscuring early-warning signals. From a SWOT perspective, these dynamics mean that AI-enabled systems can become new points of failure in *Salmonella* biosecurity architectures unless robust cybersecurity, redundancy, and incident-response plans are implemented from the outset [129,138,145,146,147,148].

***Regulatory acceptance, explainability, and institutional inertia.*** Regulatory analyses from FAO, EFSA, and national authorities converge on the point that, although AI shows clear potential for food safety and risk assessment, competent authorities are not yet comfortable basing official controls, certification, or risk categorisation schemes on opaque, non-explainable models. Instead, they advocate for human-centric, transparent, and auditable AI, with risk assessors and competent authorities maintaining responsibility for decisions [149,150,151]. Under this emerging paradigm, ML-derived “*Salmonella* risk scores” are likely to be accepted mainly as tools for prioritisation or decision support rather than as substitutes for statutory sampling frequencies, microbiological criteria, or official tests unless their logic is clearly documented and independently validated [149,151]. If regulatory frameworks and technical guidance do not evolve to incorporate explainable AI into official control schemes, sophisticated *Salmonella*-focused models may remain academic prototypes or internal industry tools rather than being formally integrated into control programmes for poultry, cattle, and pigs [129,138,145,146,147,148,152,153].

## 4. Conclusive Remarks

In conclusion, farm management practices that reduce *Salmonella* include strict biosecurity measures (such as source screening and wildlife control), maintaining good hygiene in housing and feed and water systems, minimising stress during handling, and the careful use of interventions such as vaccines or probiotics. Although cattle are less often the focus of *Salmonella* control programmes than poultry or swine, the core principles remain the same: preventing the pathogen’s entry and spread ultimately lowers the risk of contamination in carcasses and milk and reduces the burden on abattoirs and dairy plants. The integration of AI and machine learning into *Salmonella* management must be approached with balance, recognising both their transformative potential and their limitations. Ongoing collaboration among technology developers, regulatory bodies, and end users will be vital to ensure that these tools are designed and implemented transparently and fairly and remain aligned with public health goals. Continuous investment in training, infrastructure, and independent validation will also help bridge the digital divide and sustain confidence in both traditional and digital food safety methods.

## Data Availability

The original contributions presented in the study are included in the article, further inquiries can be directed to the corresponding author.
